# Initiation and Development of a Toxic and Persistent *Pseudo-nitzschia* Bloom off the Oregon Coast in Spring/Summer 2015

**DOI:** 10.1371/journal.pone.0163977

**Published:** 2016-10-12

**Authors:** Xiuning Du, William Peterson, Jennifer Fisher, Matt Hunter, Jay Peterson

**Affiliations:** 1 Cooperative Institute for Marine Resources Studies, Hatfield Marine Science Center, Oregon State University, Newport, Oregon, United States of America; 2 Northwest Fisheries Science Center, National Oceanic and Atmospheric Administration-Fisheries, Newport, Oregon, United States of America; 3 Shellfish Program, Oregon Department of Fish and Wildlife, Astoria, Oregon, United States of America; 4 Office of Science and Technology, National Oceanic and Atmospheric Administration-Fisheries, Silver Spring, Maryland, United States of America; Stony Brook University, UNITED STATES

## Abstract

In spring/summer 2015, a toxic bloom by the diatom *Pseudo-nitzschia* (PN) occurred along the west coast of the United States which led to closures of the harvest of razor clams and Dungeness crabs. Twice monthly observations of temperature, salinity, nutrients, chlorophyll and phytoplankton species composition allowed us to track oceanographic conditions preceding and during the development of the bloom. PN cells were first detected during late winter 2015. A PN bloom was initiated following the onset of coastal upwelling in mid-April; subsequent peaks in May and June were sustained by episodic upwelling events and reached magnitudes of 10^5^ cells/L and 10^6^ cells/L, 40% and 90% of the total diatom abundance, respectively. The bloom temporarily crashed in July due to a lack of upwelling, but PN cells increased again in August due to a resumption of upwelling, albeit with lower magnitude. Macronutrient conditions prior to this bloom likely played a critical role in triggering the bloom and its toxicity (particularly silicic acid limitation stress). Nutrient stress preceding the toxic bloom was related to two oceanographic events: an anomalously warm and thick water mass that occupied the northern North Pacific from September 2014 through 2015 leading to a highly-stratified water column, and the drawdown of nitrate and silicic acid during an unusually intense winter phytoplankton bloom in February and early March 2015.

## Introduction

A toxic diatom *Pseudo-nitzschia* (PN) bloom occurred in spring/summer 2015 in continental shelf waters along the west coast of the United States resulting in the closure of razor clamming (*Siliqua patula*) and crabbing (*Cancer magister*) due to contamination from a neurotoxin domoic acid (DA) produced by PN species. This is the first time the Dungeness crab fishery in Oregon closed due to this biotoxin concern [1]. High levels of DA were also detected in anchovies and sardines and led to deaths or sickness of seabirds and marine mammals, similar to reports from past toxic PN events [2, 3]. Scientists ranked this toxic bloom as the largest spatially (extending from California to Alaska) with the longest duration of any toxic event in at least the past 15 years [4].

PN blooms occur commonly in the coastal waters of the California Current, but not all blooms become toxic. Coastal upwelling has been well recognized as a driver controlling the annual initiation of PN blooms (both toxic and non-toxic) in spring and for sustaining multiple bloom cycles throughout the upwelling season [5–7]. Upwelling controls PN blooms by providing nutrients to the upper mixed layer, by regulating turbulence of the water column and possibly by regulating cell dispersal to remote regions. Regional features, such as topography and circulation patterns [7–12] can also affect the magnitude, duration and transport of PN blooms.

A diverse set of potential mechanisms have been explored to explain why PN species produce DA [13–17]. Among these, macronutrient (silicic acid and/or phosphate) and trace metal limitations (e.g. iron) have gained the most attention; however, support for these mechanisms has not always been consistent among regions [8, 9]. Silicic acid and/or phosphate limitations appear to be the primary triggers of toxic events.

In the coastal upwelling system off Oregon, toxic PN events that occurred in spring/early summer of 2005 and 2010 [12, 18] coincided with the delayed onset of the annual upwelling season and may have resulted from physiological stress associated with nutrient limitation that in turn induced DA production. However, data on nutrient concentrations in relation to toxicity were lacking in those studies. While many hypotheses have been reported to explain how macronutrient-limited conditions could interfere with the normal physiological functions of PN, the exact thresholds of nutrient regulation required to stimulate the genetic control of DA production are largely unknown [14]. As a result, toxic PN blooms are still difficult to predict in rapidly changing upwelling ecosystems.

Past toxic PN events along the west coast of United States have occurred most often during warm ocean conditions associated with the warm phase of the Pacific Decadal Oscillation (PDO) and/or El Niño Southern Oscillation (ENSO) events: fall 1991 [19], summer/fall 1998 [3, 20], summer 2002 [21], summer/fall 2004 [11], spring 2005 [22] and spring/ summer 2010 [18, 23]. The toxic event in 2015 also occurred during warm ocean conditions, but in this case, it was associated with an anomalously warm water mass (named “the Blob”) that was initiated during winter 2013–2014 in the Gulf of Alaska [24, 25], subsequently spread throughout the northern North Pacific, and persisted through at least spring 2015 [26–28]. The specific processes leading to this toxic and persistent bloom have not yet been investigated, and thus are the objectives of this study.

**Fig 1:**
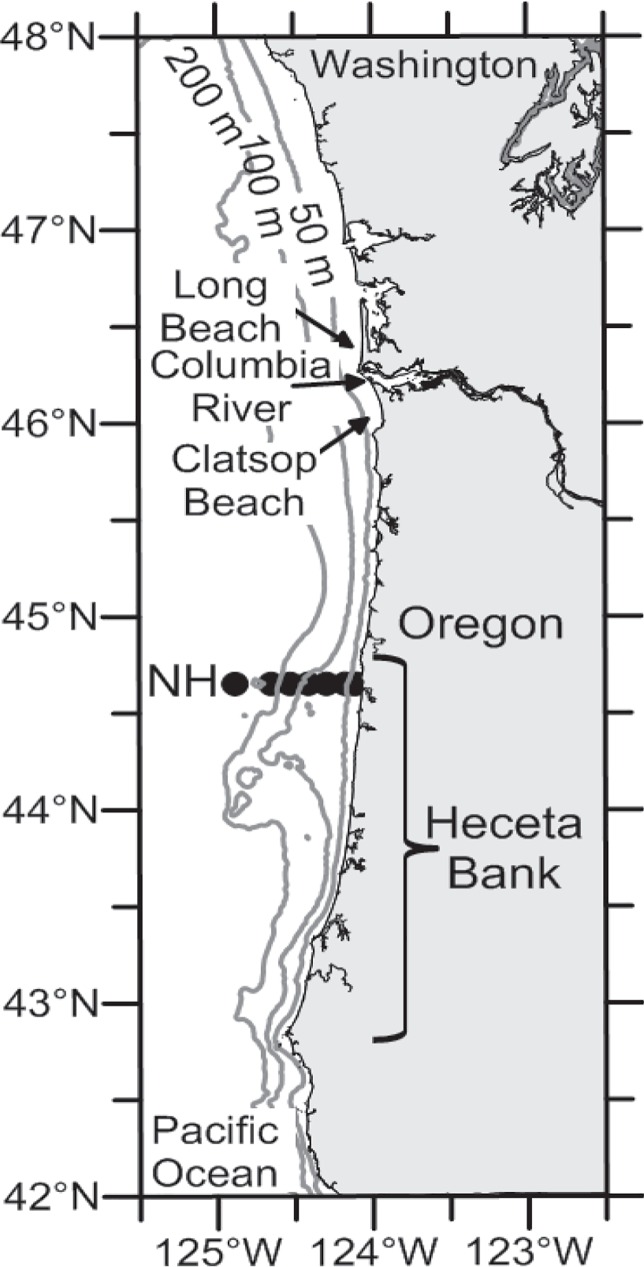
Shipboard sampling stations. Seven stations (black dots) are located sequentially from nearshore to the continental slope along the Newport Hydrographic (NH) line, central Oregon.

Given the extensive research on PN, one might expect to find a common driver accounting for all regional PN blooms and for triggering toxicity in the broader California Current upwelling system, however the exact mechanism(s) remain elusive. Here we use a unique time-series of wind, hydrographic, nutrient and phytoplankton species data collected off central Oregon (Fig 1) prior to and during the unprecedented PN bloom in 2015 to describe the environmental conditions and bloom dynamics and to explore the regional factors that might have contributed to the initiation and toxicity of the bloom. We suggest that the anomalously warm ocean conditions along with a phytoplankton bloom in winter 2015 may have been the proximate causes of this toxic and persistent bloom through preconditioning coastal waters of the California Current favorable for the explosive growth of PN in spring/summer.

## Materials and Methods

### Sampling

Twenty cruises were conducted from January to August of 2015 along the Newport Hydrographic (NH) transect off central Oregon (44.6°N), located at the northern end of Heceta Bank (Fig 1). The nearshore stations (NH1, NH3 and NH5) were always sampled at least biweekly; offshore stations (NH10, NH15, NH20 and NH25) were sampled at least monthly. Station numbers indicate distance in nautical miles, equivalent to 1.8 to 46 km from shore. No specific permissions are required for our study locations and sampling activities.

At each station, vertical profiles of temperature, salinity and fluorescence were measured from surface to within a few meters above the sea floor with a Seabird SBE 25 CTD and Wetlabs fluorometer. Surface whole water samples were analyzed for nutrients and chlorophyll *a* [29]. Surface water (125ml) for *Pseudo-nitzschia* (PN) cell counts and phytoplankton community composition was fixed with acid Lugol’s solution (2% final concentration) immediately after collection. The procedures for phytoplankton species identification and enumeration were consistent with previous work [30]. PN cells were counted as three universally accepted size categories [21] using a light microscope: the ‘wide group’ species *P*. *australis/fraudulenta/heimii*, ‘medium group’ *P*. *multiseries/pungens* and ‘thin group’ *P*. *pseudodelicatissima/delicatissima*.

### Domoic acid in razor clams

The concentration of domoic acid (DA) in razor clams was used as a proxy for the toxicity of PN blooms. The State of Oregon Department of Agriculture tests DA in shellfish tissues frequently and announces shellfish closure if DA concentration exceeds the regulatory limit of 20 ppm. Concentrations of DA in razor clam tissues are tested on the central (44.6°N, near Newport) and northern (46°N, Clatsop beach) Oregon coasts, (http://www.oregon.gov/oda/programs/foodsafety/shellfish/pages/shellfishclosures.aspx), and by the State of Washington at Long beach (46.5°N) in southern Washington (http://wdfw.wa.gov/fishing/shellfish/razorclams/domoic_levels.html). These DA data were used to examine timing and spatial synchrony of the effects of the 2015 PN blooms on razor clams (Fig 1). We do not have data on dissolved or particulate DA in whole water samples but a previous study [18] off Oregon found that DA levels in razor clam are a good indicator of toxic PN events.

### Upwelling and sea surface temperature

Daily wind stress observed at Newport Oregon (http://damp.coas.oregonstate.edu/windstress/index.html) was used to determine the timing of the spring transition, magnitude of upwelling, and periods of active and relaxed upwelling events. Sea surface temperatures (SST) monitored hourly at NOAA buoy 46050 (44.64°N, 124.50°W; 20 nautical miles west of Newport, referred to as station NH20) (http://seaboard.ndbc.noaa.gov) were used to compute daily averages of SST. Daily SST anomalies were calculated from January to August 2015 by removing the daily climatological mean using the base period of 1991–2014. The SST gaps between March 10–15 and April 10–21 were filled with data from a nearby buoy 46094 (44.64°N, 124.50°W; 10 nautical miles west of Newport, at NH10). Missing SST data from March 16 through April 9 due to buoy malfunction were assumed to be similar to the days preceding and following this gap, which was reasonable due to continuously southerly winds during this period.

## Results

### Wind, temperature, nutrients and chlorophyll *a*

**Fig 2:**
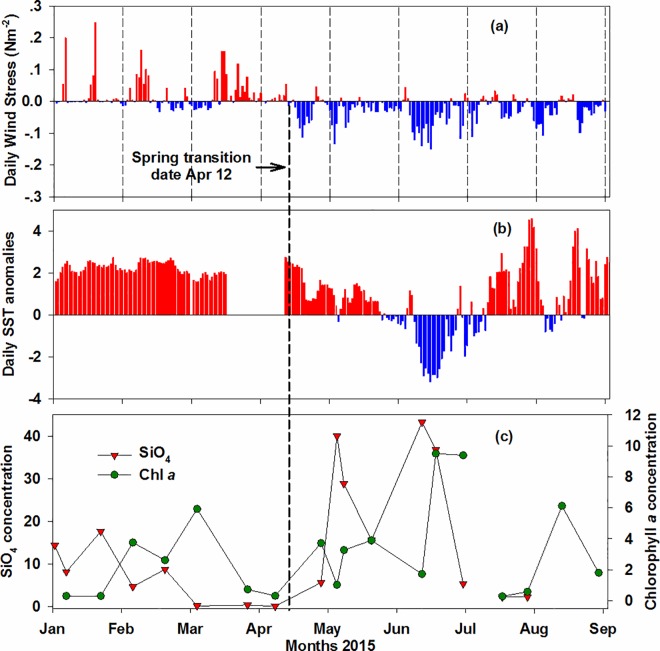
Environmental conditions observed off Newport, Oregon. The x-axis (January through August, 2015) is shared by the three panels. The dashed vertical black line across panels indicates date of the spring transition. (a) Daily alongshore wind stress (N m^-2^). Red bars indicate southerly winds (downwelling/relaxation of upwelling) and blue bars indicate northerly winds (upwelling). (b) Daily sea surface temperature anomalies (colored bars, SST °C) from the buoy 46050 located at station NH20, 37 km offshore Newport. (c) Surface silicic acid (μM) and chlorophyll *a* (μg/L) concentrations measured during shipboard samplings at station NH5, 9 km from shore.

Winter conditions off the Oregon coast in January and early February 2015 (Fig 2A and 2B) were characterized by a lack of southwesterly storms and warm sea surface temperatures (SST, +2°C anomalies). Only two brief storms occurred in January and one in early February, otherwise winds were unusually calm. During this period, nitrate (NO_3_) and silicic acid (SiO_4_) concentrations were moderate (Table 1). Winds became northerly in mid-February and persisted through early March. Skies were clear, promoting a winter phytoplankton bloom with Chl *a* ranging between 2.6 and 5.9 μg/L (Fig 2C). The winter bloom led to a severe depletion of NO_3_ and SiO_4_ extending from March through early April, 0.3–0.4 μM and 0.01–0.3 μM, respectively. These concentrations were less than 10% (NO_3_) and less than 2% (SiO_4_) of climatological concentrations of ~ 4μM and ~ 11μM in March-April, respectively (2001–2014). Phosphate (PO_4_) concentrations were relatively high; both N:P and Si:N ratios were extremely low, < 0.3 and < 1, respectively (Table 1).

**Table 1:** Temperature and nutrients at the nearshore station NH5 in 2015.

Dates	T-20	NO_3_	SiO_4_	PO_4_	NH_4_	N:P	Si:N
1-Jan	12.1	6.1	14.3	0.8	0.2	7.9	2.3
6-Jan	12.5	4.1	8.0	0.8	0.3	5.5	1.9
21-Jan	12.5	5.2	17.6	0.7	0.7	8.6	3.0
4-Feb	na	0.3	4.6	0.5	0.4	1.4	7.1
18-Feb	12.5	1.7	8.6	0.6	0.2	3.3	4.6
4-Mar	11.7	***0*.*4***	***0*.*1***	***2*.*4***	***0*.*2***	***0*.*3***	***0*.*2***
26-Mar	12.3	***0*.*4***	***0*.*3***	***5*.*8***	***0*.*1***	***0*.*1***	***0*.*8***
7-Apr	12.0	***0*.*3***	***0*.*01***	***1*.*4***	***0*.*1***	***0*.*3***	***0*.*04***
27-Apr	10.3	***0*.*06***	5.6	*0*.*4*	0.1	***0*.*3***	49.2
4-May	8.6	23.8	40.1	2.0	0.2	11.9	1.7
7-May	9.1	16.2	28.8	1.5	0.8	11.7	1.7
19-May	9.3	6.4	15.6	0.8	0.5	8.8	2.2
10-Jun	8.3	25.6	43.3	3.3	0.7	7.9	1.6
16-Jun	7.9	21.1	36.8	1.8	0.2	12.2	1.7
28-Jun	9.0	3.0	5.3	0.5	0.1	6.6	1.7
3-Jul	9.9	Na	na	na	na	na	na
15-Jul	12.5	0.3	2.2	0.4	0.5	0.9	6.1
26-Jul	13.4	0.0	2.2	0.3	0.2	0.2	33.1
10-Aug	10.2	0.6	2.0	1.0	1.2	1.8	1.1
26-Aug	9.9	0.2	0.7	0.5	0.2	0.7	2.1

T-20 (°C) represents an average temperature of the upper 20m water column. Listed nutrient concentrations (μM) represent surface water conditions. NO_3_: nitrate; SiO_4_: silicic acid; PO_4_: phosphate; NH_4_: ammonium; N:P ratio: (NO_3_+NH_4_): PO_4_; Si:N ratio: SiO_4_: (NO_3_+NH_4_). Numbers in bold italics indicate dates when severe N and Si limitation were observed. The middle line segregates cruises done before and after spring transition. na: data not available.

The onset of the upwelling season (also known as the spring transition), marking the transition in winds from southerly downwelling conditions to a dominance of northerly upwelling favorable conditions, began on April 12. Northerly winds were light initially, stronger during April 16–22 and then reversed to southerly causing a relaxation of upwelling. During this early phase of the upwelling season, the ocean remained anomalously warm with offshore SST cooling only by 1°C (Fig 2B) while water column nearshore cooled by ~ 2°C (T-20, Table 1) indicating that cool nutrient-rich water was only occupying waters of the inner-mid shelf. Nutrients were replenished to the upper water column during this first strong upwelling event (April 16–22) and a diatom bloom (including PN) developed, increasing Chl *a* concentration (Fig 2C) to 3.7 μg/L on April 27. This bloom resulted in severe depletion of nutrients again, drawing NO_3_ and PO_4_ down to 0.06 μM and 0.3 μM by April 27, respectively (Table 1).

The northerly winds resumed on April 28, and persisted through May 7 (Fig 2A) but then became relatively weak, resulting in continuously weak upwelling for the remainder of the month. The strongest northerly winds of the year were observed in June resulting in the lowest temperatures both offshore (-3°C anomaly, Fig 2B) and nearshore (T-20 < 8–9°C, Table 1) indicating that upwelled water was found across the entire continental shelf. During the relatively consistent period of upwelling in May and June, the macronutrient concentrations nearshore followed the classic pattern in an upwelling system with lower concentrations during the weak or relaxed upwelling events and higher concentrations following strong upwelling events. At no time in May or June did NO_3_ concentrations fall below 3 μM or SiO_4_ below 5 μM. The ratios of N:P ranged between 7.9–12.2 and Si:N between 1.6–2.3 (Table 1).

In contrast, after early July northerly winds were mostly weak and temperatures were high both nearshore with T-20 > 13°C and offshore with the highest SST anomalies (+ 3–4°C). Nitrate concentrations dropped to < 1 μM and silicic acid, 2.2 μM. In August, northerly winds were stronger than July (but weaker than June) causing slight cooling nearshore (T-20 < 11°C), but offshore SST anomalies remained as high as in July indicating again that upwelled waters were not extending very far offshore. In August, NO_3_ concentration were between 0–0.6 μM; SiO_4_ was slightly higher, 0.7–2.2 μM (Table 1).

### PN bloom and DA

**Fig 3:**
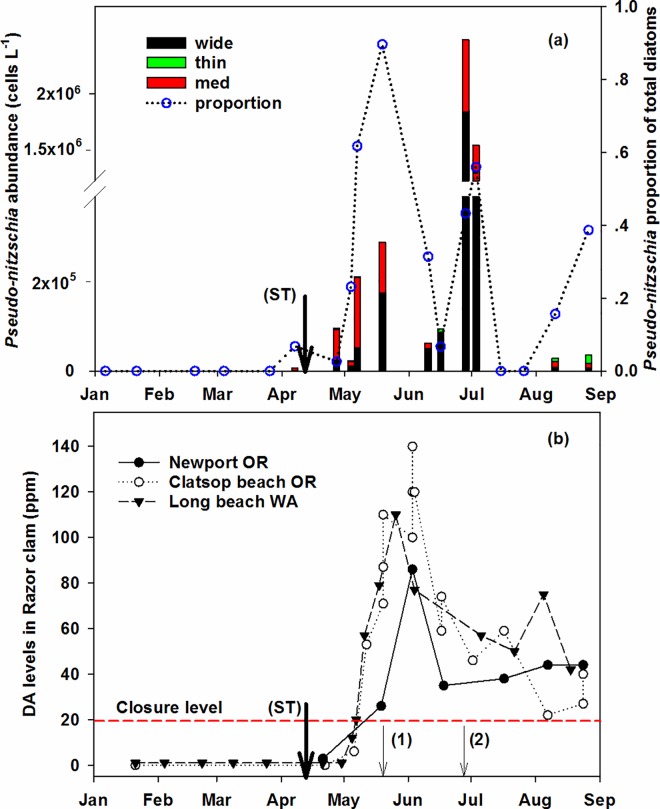
In situ observations of PN bloom and domoic acid in 2015. (a) The bloom initiation and development observed at the nearshore station NH5. Bars stacked by the three sized PN groups indicate cell abundances (left y-axis). Circles connected by dotted lines represent proportions of PN (blue circles, right y-axis) in the total diatom abundance. (b) Domoic acid (DA) concentrations (ppm) in razor clams. The dashed red line indicates the 20ppm closure threshold for razor clam harvest. Downward arrows mark the date of spring transition (ST) and timing of the first (1) and second (2) peaks of PN bloom in May and June, respectively.

Past DA events [2, 3, 6–8] are often associated with the three differently-sized PN cells, therefore, it is more informative to present data separately on the abundance of the three PN groups. The larger PN cells (wide and medium) were dominant throughout the major bloom period, and thus in the following text PN refers to the larger morphs of PN. PN cells were first observed on March 26 (380 cells/L) at the most nearshore station NH1, and by April 7 prior to the spring transition, increased up to ~7000 cells/L at NH5 (Fig 3A). The medium group was relatively more abundant in April and early May but the wide group gradually became more dominant from later May.

After the spring transition, PN abundance increased to 10^5^ cells/L in late April but contributed only a low percentage (2.5%) of the total diatom abundance (Fig 3A). The first reported DA concentration of the year in razor clams near Newport was from April 21 (2.9 ppm), a few days before the significant increase in PN numbers. The May 4 sampling was conducted immediately following a brief episode of strong northerly winds (April 30 to May 3) and PN abundance had decreased somewhat. Following the next episode of strong northerly winds (May 7–12), PN abundance increased again. During early May, although there were no DA measurements in clams at Newport, the first increases of DA were reported at the two northern locations, Clatsop beach (northern OR) on May 6 (6.1 ppm) and Long beach WA on May 5 (12 ppm) (Fig 3B). On May 14, the entire Oregon coast was closed to razor clamming in response to the thriving and toxic PN bloom. By May 19, the first bloom peaked at Newport along with a dramatic increase of PN percentage from 23% to 90% of total diatoms, indicating the formation of a mono-specific PN bloom. Meanwhile, DA in clams was measured as 26 ppm on that day and reached its highest level (86 ppm) by early June at Newport. At a similar time, DA reached maximum concentrations at Clatsop beach (140 ppm on June 3) and Long Beach (110 ppm on May 26). The bloom began to decay in mid-June inferred from the declines of both the abundance and proportion of PN, and at the same time, DA levels in clams decreased by half at the three locations (Fig 3A and 3B).

A second PN bloom peak was observed on June 28 and cell abundance remained high in early July with a higher magnitude (10^6^ cells/L) than the first peak. The percentages of PN cells were about half of total diatom abundance. Species richness in the diatom community was greater during the second peak than the first peak, 20–22 vs. 4–7 species respectively (S1 Table). DA in clams did not increase at this time even though PN abundances were at a maximum.

PN cells were absent in mid-late July, a time when the phytoplankton community composition shifted from diatom dominance to dinoflagellate-ciliate co-dominance (S1 Table). However, PN cells recurred following a resurgence of stronger upwelling in early August, but a bloom did not form until the end of August albeit with a low magnitude and an increased proportion of the thin PN group (Fig 3A). In July and August, DA in clams either maintained a constant level (~40 ppm) near Newport or slowly decreased in northern OR and southern WA.

**Fig 4:**
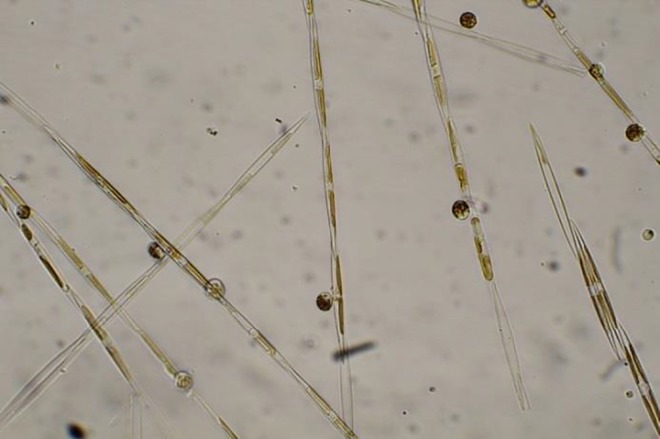
An image of the large celled PN.

Active sexual reproduction was only observed during the first bloom peak in May when empty parental frustules (from the large celled PN) and gametes/zygotes-like were observed in a live water sample (Fig 4). Fewer sexually reproductive cells were present in other samples taken from April to August.

### Onshore-offshore patterns of PN blooms

Cross-shelf dispersal of PN cells was related to the event-scale upwelling/relaxation cycles. During upwelling relaxation events, PN cells were mostly aggregated in the nearshore waters and gradually decreased towards offshore. During persistent and stronger upwelling events, PN abundance was higher in offshore waters. For example, > 10^5^ cells/L abundance was measured at the continental slope (NH25) in early May, and the bloom extended out to at least 60 km from shore (S1 Fig). During the weak and intermittent upwelling period, the densest center of abundance was close to shore, with a more gradual decrease seaward.

### Vertical patterns of temperature and fluorescence

In January and February 2015, the unusually warm (> 12°C, + 2°C anomaly) water mass occupied the upper 50–80 m waters of the water column off Newport. The northerly winds in early March cooled the upper layer slightly, but soon downwelling returned leading to a thick layer of warm water over the upper 40–50 m of the continental shelf through early April (S2 Fig).

**Fig 5:**
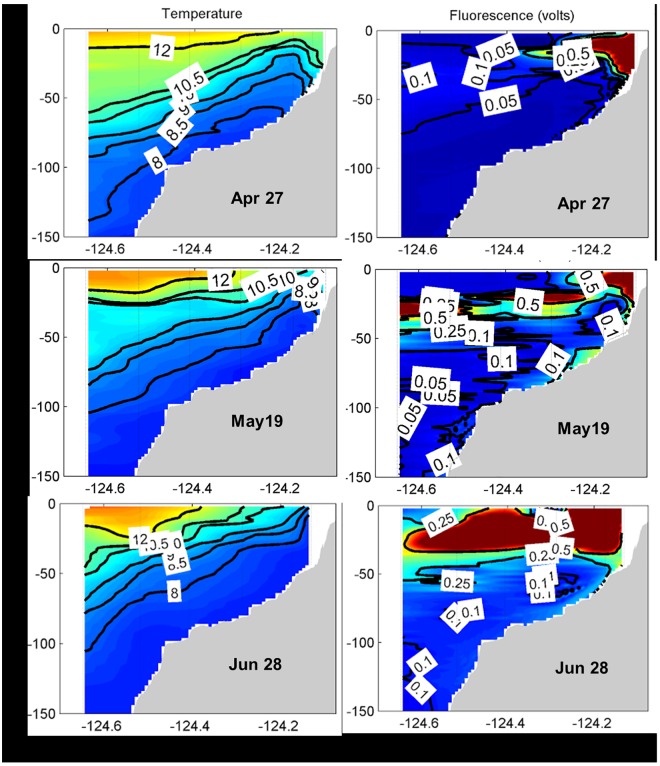
Vertical contours of temperature (°C, left) and chlorophyll fluorescence (volts, right). Contour plots on three samplings dates of April 27, May 19 and June 28 (the major PN bloom period in 2015) are shown for the Newport Hydrographic transect (44.6°N).

After the spring transition, the water column showed the upward titling of isotherms nearshore (Fig 5, left panels) indicating upwelling. Cold water (< 10°C) upwelled near the surface in the inner- and mid-shelf, and warm water (> 12°C) was displaced to outer-shelf and slope. Fluorescence plots illustrate contrasting dense layers from April to June (Fig 5, right panels). On April 27, a mixed-species bloom assemblage occupied the entire water column at the inner shelf as indicated by high fluorescence. During the first PN bloom peak on May 19, the dense layers of high fluorescence may have been composed entirely of toxic cells (recall 90% contribution of PN in the sea surface samples), and may have gradually descended from surface in inner shelf waters to depths of 20-30m in slope waters. During the second PN bloom peak on June 28, the high fluorescence signal in both shelf and slope waters indicates a much higher bloom magnitude (mixed-species) than in May, as noted previously. PN cell abundance was 43% of total diatom abundance (S1 Table).

## Discussion

We hypothesize two events, which preceded the initiation of the toxic PN bloom in April 2015, may provide *proximate* explanations for why this bloom occurred and why it was toxic. The first event is related to “the Blob”, a mass of anomalously warm water nearly 100 m thick that evolved in the Gulf of Alaska in winter 2013/2014 and spread throughout the northern North Pacific by the summer of 2014 with surface temperature anomalies in excess of +4°C [24, 26–28]. After the Blob intruded on the Oregon shelf in September 2014, it occupied the upper 50 to 80 m of the water column through early April 2015 (S2 Fig), causing strong stratification, deepening of the thermocline (and nutricline) and low nutrient concentrations each of which has been identified by others as key variables that contribute to the increase of harmful algal bloom in response to ocean climate changes [17]. Since historical closures of razor clam harvest due to DA events in Oregon have mostly coincided with other warm water events associated with ENSO and/or warm phase of the PDO, as in 1991 [31], 2002–2005 [12] and 2009–2010 [18], we suggest that the presence of warm water on the shelf from mid-September 2014 through winter 2015 associated with the Blob, along with the increased stratification and reduced nutrient concentrations may have provided a proximate cause for the initiation of PN bloom in April.

The second event that could have been a proximate cause of the toxic bloom is the winter phytoplankton bloom from February to early March 2015. Such blooms can occur off Oregon in late winter or early spring but only in years when winds are light and skies are clear [32]. The winter bloom in 2015 was the largest that we have observed in 20 years off Newport, and was dominated by diatoms (S1 Table). Following the winter bloom, a near-complete depletion of nitrate and silicic acid persisted for at least one month prior to the spring transition, thus leading to stressful growth conditions for diatoms (including PN). During the months of January-April, nitrate and silicic acid concentrations averaged 62% and 73% of climatology in January, respectively, but were uncommonly low, ≤ 10% and 2% of climatology, respectively, from February through early April. Therefore, we suggest that the combined effects of an anomalously warm and stratified ocean along with the winter diatom bloom contributed to nutrient stress, and resulted in a “preconditioning” of continental shelf waters for the toxic PN bloom that was initiated off Oregon in April 2015. The timing of key events related to the PN bloom and environmental conditions before and during the PN blooms are summarized as a time line in S2 Table.

### The PN bloom initiation and development

Local-scale forcing from coastal upwelling and associated nutrient conditions likely provide the *ultimate* explanation for development of a toxic PN bloom. After the spring transition in mid-April, nutrients were replenished and PN along with other diatoms responded quickly to the enhanced nutrient supply, thus a large diatom bloom was formed. This bloom subsequently reduced nitrate but this time, to its lowest record (0.06 μM in late April) during our study. This, the first bloom following the onset of the upwelling season, must have, once again, imposed a significant stress on surviving diatoms. After northerly winds resumed in May, high concentrations of nutrient were brought to the upper mixed layer, and another diatom bloom developed but this one was dominated by only a small subset of taxa including PN, *Skeletonema costatum* and *Nitzschia* (S1 Table) suggesting that only these species (along with PN) survived the extremely low nutrients in late April and thus were also able to respond quickly to the sudden infusion of high nutrients in early May. Such a growth strategy in association with upwelling pulses has been demonstrated in previous studies [13]. PN are thought to be better competitors under turbulent conditions in the upwelling system also because they could sink out of the euphotic zone quicker which would help them avoid being transported offshore and lost [2]. These strategies may allow PN to out-compete those diatoms which need a more stable and stratified water column to form blooms [33].

After the two brief strong upwelling episodes in early May, weaker and more intermittent northerly winds prevailed, promoting a mono-specific PN bloom (90% of all diatoms on May 19). Macronutrient concentrations and ratios of Si:N and N:P in May fell in the normal range compared to their long term records for the upwelling season [34]. However, despite “normal” nutrient concentrations, some of the diatoms that occur commonly during upwelling, e.g., *Chaetoceros* and *Thalassiosira* [30] were lacking and the phytoplankton community was rather simplified with low species richness. We suggest the unusually low diversity of the phytoplankton community was the result of rapid transition of nutrient concentrations from severe depletion to repletion and upwelling-associated turbulence (dispersal and loss to offshore) during the end of April to early May, conditions that may have favored only a handful of species (S1 Table).

The phytoplankton community and harmful algal blooms have been monitored off Newport since 2001 [5, 35]. From this dataset, it is clear that the lengthy persistence of the PN bloom in 2015 is unprecedented. In the past, the most persistent bloom of the large celled PN was in 2010, and had a duration of about 2 ½ months, extending from the end of June to early September [18]. Other past PN blooms (both toxic and non-toxic) off Newport persisted for no more than a month, but often with several short PN blooms occurring during a given upwelling season.

The extended PN bloom in 2015 may have been aided by unique aspects of cross-shelf circulation associated with the coastal upwelling process. That is, during active upwelling events when offshore Ekman transport was strong, while PN cells were transported offshore, they also sink into deeper water that became the seed population wide-spread over the shelf and slope waters. With a relaxation of upwelling, PN in surface waters would be carried back to shore. On the other hand, with the next bout of active upwelling, PN cells in deeper waters would be transported shoreward with the shoreward-moving upwelled water and eventually returned nearshore. This idea was first proposed for “re-seeding” of copepod populations in the Oregon upwelling zone [36, 37]. Such a mechanism may also account for the resurgence of the PN bloom in August after a complete absence of PN cells for several weeks in July. Thus, as suggested by Trainer et al. [38], the vertical movement of PN cells and recirculation by the upwelling process may have facilitated persistence of PN blooms. The movement of PN species was attributed to subsurface layer formation in stratified waters which in turn would provide potential seed populations for future blooms [39]. In our study, the subsurface layers indicated by chlorophyll fluorescence were clearly seen from the shelf waters to the slope during the mono-specific PN bloom in May (Fig 5). The idea for a subsurface origin of seeds offshore leading to PN blooms nearshore was recently demonstrated in the southern California Bight by Seegers et al. [23] who incorporated glider and Environmental Sampling Processor observations. Their near-real time data confirmed the direct advection of the PN population shoreward through subsurface layers.

### DA induction

Even though dissolved or particulate DA in the water was not measured during cruises, the frequent tests of DA in razor clams overlapped well with the time period of the PN bloom initiation and development which aids in the identification of the period when PN cells became toxic and of the potential factors inducing DA. Although the first detection of DA in razor clams on April 21 near Newport (central Oregon) was much lower (2.9 ppm) than the regulatory limit of 20 ppm, it indicates that PN cells were already toxic before that date. Recall that a few PN cells were found in the inner shelf in late March, and were present in small numbers in early April. PN abundance increased around the time of spring transition (April 12) and continued to increase during the strong upwelling event of April 16–22. Onshore and northward transport patterns were dominant during April 10–13 and again on April 23–27 as shown in the surface current maps near Newport (http://agate.coas.oregonstate.edu/data/codar_np.html). This suggests that the periods of April 10–13 and April 23–27 were times when toxic cells were brought to the very nearshore zone where razor clams live, and later led to the first occurrence and continuous increase of DA in razor clams.

Meanwhile, periods of nutrient stress preceded and/or were coincident with the window of DA induction. For one month prior to the spring transition, silicic acid concentration (0.01 μM) and Si:N ratio (0.04) were the lowest values for the year 2015. Either silicic acid or phosphate limitation has long been considered as triggering PN physiological changes and inducing DA [14]. In this study, phosphate was clearly not limiting whereas the persistently low Si:N ratios, and equally, Si stress, seemed to be the most likely trigger. The ratio of Si:N < 2 was suggested to induce DA in the Santa Barbara Channel and Si:N < 1 was confirmed to cause DA production [40]. Sufficient nitrogen supply is essential to the synthesis of DA molecules [15], thus we hypothesize that this requirement contributed to setting up the time window when DA was produced at around the same time as the date of spring transition. PN cells might have turned to other nitrogen forms such as using dissolved organic nitrogen to synthesize DA [41] before the spring transition. Another possibility of nutrient stress triggering DA production is iron limitation, however, previous work has shown that iron concentrations off Oregon are not limiting to phytoplankton growth [42].

Three factors were associated with the rapid increase of DA in razor clams from late May to early June coinciding with the increasing trend of PN bloom magnitude. The first factor was related to the mono-specific bloom formation. This alone could accelerate the bioaccumulation of DA by razor clams compared to when a mixture of abundant diatom species is available because a higher concentration of other diatoms would dilute toxin intake in clams feeding on these non-toxic prey [2]. A second factor contributing to the rapid increase of DA concentration in razor clams is the active sexual reproduction (auxo-sporulation) that was observed during the peak of the bloom in late May, and the sharp increase of DA in razor clams in early June followed soon after this bloom peak. A previous study [43] showed evidence that an increasing portion of newly sexually reproduced PN cells coincided with the highest DA level. That seems to indicate some potential link between PN sexual reproduction and DA production. Thirdly, upwelling was intermittent during mid- and late May and thus toxic PN cells were not transported offshore, rather were retained close to razor clam beds. The continuous exposure to the toxic cells would have led to the sharp increase of DA level in clams.

Following the PN bloom peak in late May and DA peak in early June, DA concentrations in clams decreased by half, and remained at lower but steady levels through August. This too may have been influenced by three factors: (i) the slow depuration rate of DA by clams [19], (ii) reduced concentrations of DA in PN cells and (iii) offshore transport of PN cells due to stronger upwelling observed throughout June. Had we measured particulate DA we may have been able to evaluate the importance of the second factor. Regardless it is clear that the largest PN bloom of the year, observed in June, did not lead to further increases of DA in clams. Finally, the sudden decline of DA in clams in mid-June could also be related in part with the spawning of clams annually during May to July [44]. When they spawn, all gametes are released simultaneously resulting in a large loss of body mass, and possibly DA as well.

The important physical process contributing to DA transmission along the food chain is the contrasting roles that coastal upwelling plays in (a) bringing nutrients to the photic zone that are required by PN blooms as opposed to (b) the transport-mediated regulation of toxic PN cells availability as prey. If upwelling is strong, DA-enriched PN cells will reproduce rapidly but meanwhile would be transported away from the nearshore zone reducing the chance of razor clams feeding on toxic cells, whereas when upwelling is moderate or weak, PN cells would be more readily retained in nearshore zone, thus increasing DA loads in clams.

Although we have no data on DA toxicity per PN cell, it is clear that PN cells were toxic in May, given that razor clams only began to exceed the regulatory limit of DA concentrations (20 ppm) when the PN bloom had continued and peaked in May. It is perhaps unfortunate that we have no data in DA per PN cell, but it is clear that to understand the factors that determine DA in razor clams, one needs to know more than DA per PN cell–other factors need to be considered for this study system, including onshore transport of PN-enriched water during the relaxation of upwelling which continuously exposes razor clams to toxic PN cells, the possibility that sexual reproduction leads to higher levels of DA in PN, slow depuration rates of razor clams, and the potential for spawning by clams leading to a loss of DA in clams.

### Synchronicity of the PN blooms

Data supporting the observations that the PN bloom in 2015 was initiated synchronously along the west coast of United States can be seen by referring to Blog sites that reported on sampling from fishing piers for southern California [45], and for Monterey Bay, central California [46], along with surf-zone sampling by Washington Department of Fish and Wildlife (MH). In each case listed above, the PN bloom became a dominant feature beginning in April and extended through at least August 2015. The coastwide warm ocean condition due to the Blob effects (as described above) appears to be the most likely cause of the synchronicity feature. Alternatively, oceanic transport plays a role in spreading harmful algal blooms but is a challenging question to address due to the inability of tracking the dispersal of cells at large spatial scales. There is evidence that localized regions sequester seed stocks of PN cells due to retentive circulation patterns and serve as “hot spots” that provide the sources of seed stocks for PN blooms. The Juan de Fuca eddy area off WA and Heceta Bank off OR are two such sites in the northern California Current [16]. Both in situ sampling studies [3, 11, 12] and model simulations [47] have confirmed the idea of the formation of toxic PN blooms at “hot spots” and the subsequent spread of blooms through alongshore transport. However, it is beyond the scope of this study to evaluate the role of transport playing in connecting the bloom that we observed off central Oregon with distant regions.

## Conclusions

Our study off Newport, Oregon is among the first to document environmental conditions prior to and during the early development of the toxic PN bloom and subsequent toxicity in razor clams in the California Current in 2015. Given that this bloom was observed synchronously throughout much of the California Current, from California to Washington resulting in devastating impacts on coastal fisheries and the local economy for over half a year, synthesis of data collected by leaders of the west coast community of HAB scientists will provide an opportunity to examine the common triggers for the initiation of the bloom and possible connections across regions. Further analysis of this event may eventually allow oceanographers to have learned enough about common triggers to allow a forecast the occurrence of toxic PN blooms.

## Supporting Information

S1 Fig Cross-shelf patterns of the *Pseudo-nitzschia* bloom along the Newport Hydrographic line. (TIF)

S2 Fig Vertical contours of temperature (°C) prior to the initiation of *Pseudo-nitzschia* bloom. (TIF)

S1 Table Phytoplankton community composition prior to and during the *Pseudo-nitzschia* bloom. (DOCX)

S2 Table A time line of the key events prior to and during the *Pseudo-nitzschia* bloom in 2015. (DOCX)

S1 Text Descriptions of phytoplankton community changes following S1 Table. (DOCX)
