# Supplementary figures and images for: Initiation and Development of a Toxic and Persistent Pseudo-nitzschia Bloom off the Oregon Coast in Spring/Summer 2015
Source: PLoS One. 2016 Oct 12;11(10):e0163977. doi: 10.1371/journal.pone.0163977 (PMC5061394; doi:10.1371/journal.pone.0163977)

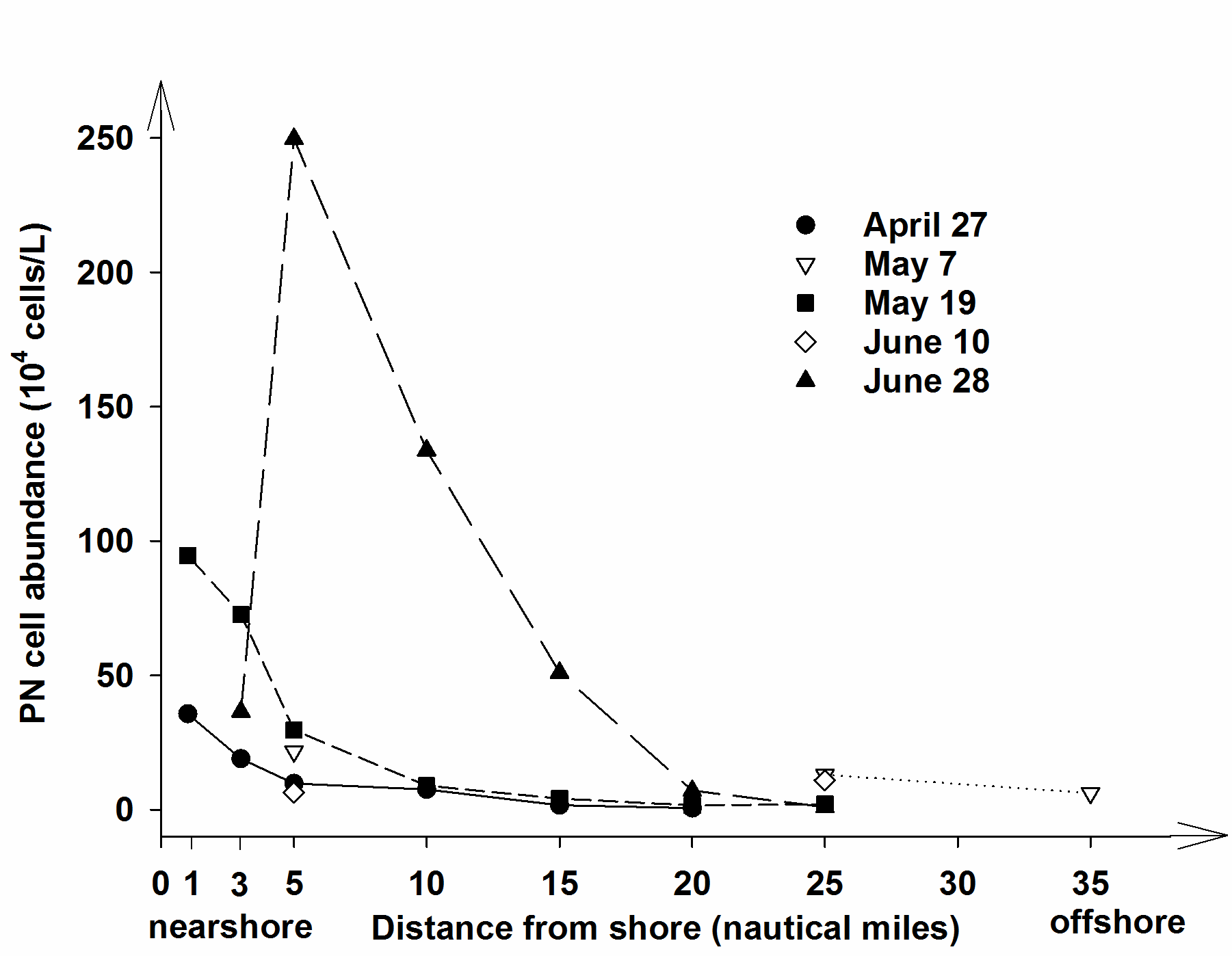

Supplement: S1 Fig — (TIF) [file pone.0163977.s001.tif]

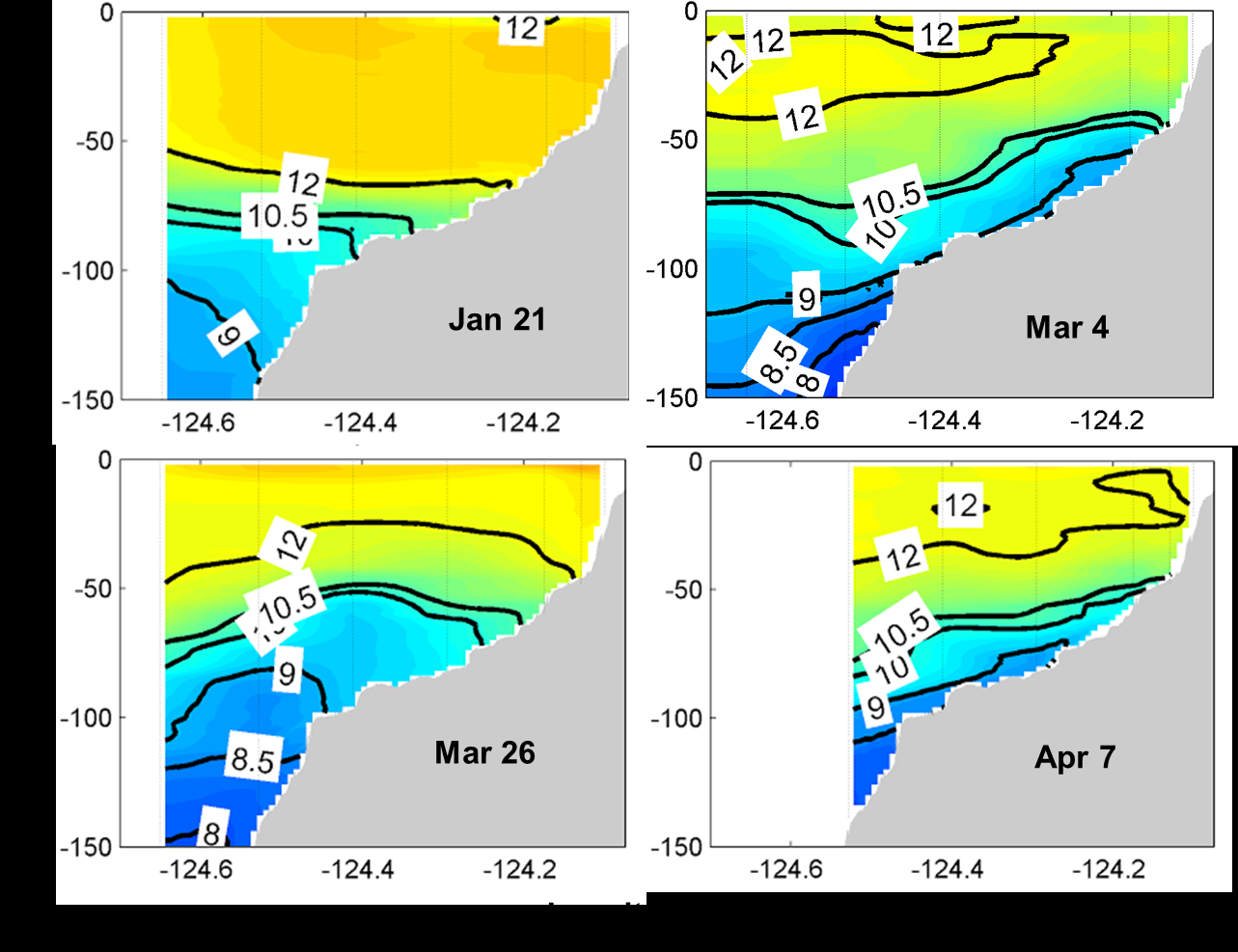

Supplement: S2 Fig — (TIF) [file pone.0163977.s002.tif]
