# Supplementary material for: Initiation and Development of a Toxic and Persistent Pseudo-nitzschia Bloom off the Oregon Coast in Spring/Summer 2015
Source: PLoS One. 2016 Oct 12;11(10):e0163977. doi: 10.1371/journal.pone.0163977 (PMC5061394; doi:10.1371/journal.pone.0163977)
Supplement: S1 Table — (DOCX) [file pone.0163977.s003.docx]

**S1 Table. Phytoplankton community composition prior to and during the *Pseudo-nitzschia* bloom**

| Dates | %PN | %Diat | %Dino | %Ciliate | S | Other dominants (abundance above 10^4^ ~ 10^6^ cells/L level) | Chl *a* |
| --- | --- | --- | --- | --- | --- | --- | --- |
| 1-Jan | 0% | 46.2% | 40.5% | 13.3% | 0 | None | - |
| 6-Jan | 0% | 58.0% | 34.1% | 8.0% | 0 | None | 0.3 |
| 21-Jan | 4.5% | 38.9% | 50.4% | 10.6% | 0 | None | 0.3 |
| 4-Feb | 0% | 96.4% | 2.8% | 0.8% | 7 | *Asterionellopsis glacialis*, (and *socialis*), *Thalassiosira* (2), *Chaetoceros debilis* | 3.8 |
| 18-Feb | 0% | 87.8% | 7.9% | 4.3% | 5 | *Thalassiosira* (3), *Nitzschia*, *Navicula* | 2.6 |
| 4-Mar | 0% | 95.6% | 3.3% | 1.1% | 4 | *Ch. debilis* (2 others), *Skeletonema costatum* | 5.9 |
| 26-Mar | 0% | 95.6% | 3.1% | 1.3% | 5 | *S. costatum*, *Ch. debilis* (2 others), *Cylindrotheca closterium* | 0.7 |
| 7-Apr | 6.8% | 86.9% | 12.3% | 0.8% | 3 | *Rhizosolenia setigera*, *S. costatum* | 0.3 |
| 27-Apr | 2.5% | 97.4% | 2.3% | 0.3% | 25 | *S. costatum*, *A. glacialis*, *Chaetoceros* (11), *Leptocylindrum minimus* | 3.7 |
| 4-May | 23.1% | 96.3% | 3.0% | 0.7% | 5 | *S. costatum*, *Nitzschia* | 1.0 |
| 7-May | 61.7% | 96.8% | 2.7% | 0.5% | 7 | *Thalassiosira subtilis*, *Nitzschia*, *S. costatum*, *Detonula pumila* | 3.3 |
| 19-May | 89.6% | 95.1% | 2.9% | 2.0% | 4 | *Eucampia zudiacus* | 3.9 |
| 10-Jun | 31.4% | 99.3% | 0.7% | 0.0% | 9 | *Ch. debilis*, *Thalassiosira* sp., *L. minimus* | 1.7 |
| 16-Jun | 6.7% | 99.4% | 0.4% | 0.2% | 18 | *Chaetoceros* (4), *Thalassiosira* (4), *A. glacialis*, *L. minumus* | 9.5 |
| 28-Jun | 43.2% | 99.4% | 0.4% | 0.2% | 20 | *Ch. debilis* (6 others), *S. costatum*, *E. zodiacus*, *Thalassiosira* (2) | 9.4 |
| 3-Jul | 56.0% | 98.4% | 1.2% | 0.4% | 22 | *A. glacialis*, *Ch. debilis* (7 others), *S. costatum*, *E. zodiacus* | - |
| 15-Jul | 0.0% | 9.4% | 45.3% | 45.3% | 0 | no diatoms; mostly dinoflagellates, ciliates and nanoflagellates | 0.2 |
| 26-Jul | 0.0% | 12.0% | 44.8% | 43.2% | 0 | no diatoms; mostly dinoflagellates, ciliates and nanoflagellates | 0.3 |
| 10-Aug | 15.6% | 75.2% | 17.1% | 7.7% | 6 | *Ch. debilis*, *Ditylum brightwellii*, *Nitzschia*, *Probosia alata* | 3.5 |
| 26-Aug | 38.6% | 66.4% | 25.7% | 7.9% | 5 | *Rhizosolenia styliformis*, *C. closterium* | 0.7 |

Phytoplankton community composition and total Chl *a* concentration (μg/L) observed in the surface water at the nearshore station NH5 from January through August 2015. The second through fifth columns indicate percent *Pseudo-nitzschia* (%PN) of total diatom abundance, and the percent diatoms, dinoflagellates and ciliates of the total of these three major functional groups. Also listed is species richness (S, for those species whose abundance was > 10^4^ cells/L). Numbers in bracket following an algal genus indicate the number of species identified and counted.
