# Supplementary material for: Initiation and Development of a Toxic and Persistent Pseudo-nitzschia Bloom off the Oregon Coast in Spring/Summer 2015
Source: PLoS One. 2016 Oct 12;11(10):e0163977. doi: 10.1371/journal.pone.0163977 (PMC5061394; doi:10.1371/journal.pone.0163977)
Supplement: S2 Table — (DOCX) [file pone.0163977.s004.docx]

**S2 Table. A time line of the key events prior to and during the *Pseudo-nitzschia* bloom in 2015.**

**Pre-*Pseudo-nitzschia* bloom**

Sep 2014-Mar 2015 Warm Blob dominates shelf hydrography

Feb 2015 Winter phytoplankton bloom 🡺(Feb – early Mar)

Mar 2015 Severe nutrient limitation (N and Si <0.4µM) 🡺 4 Mar – 7 Apr

**First P*seudo-nitzschia* bloom cycle (7 Apr-early June)**

26 Mar A few PN cells in inner shelf waters

7 Apr PN cells increased off Newport

12 Apr Spring Transition

16-22 Apr First upwelling event; nutrients replenished

21 Apr DA first noted in razor clams near Newport, central Oregon

27 Apr Severe nutrient limitation again (NO_3_=0.06µM)

PN bloom = 2.5% of total diatoms

1-3 May Second upwelling event:NO_3_=24µM, SiO_4_=40µM

4 May PN bloom = 24% of total diatoms

5-7 May Third upwelling event; NO_3_ = 16 µM, SiO_4_=29 µM

5 May DA first exceed 20ppm in razor clams at Long beach, southern Washington

6 May DA first noted in razor clams at Clatsop Beach (northern Oregon)

7 May PN bloom = 62% of total diatoms

Closure of razor clam harvest off southern Washington

9-31 May Upwelling weak; SST > +1°C anomaly, NO_3_>6µM, SiO_4_>29µM

12 May Closure of razor clam harvest off northern Oregon

14 May Closure of razor clam harvest for entire Oregon coast

19 May PN bloom = 90% of total diatoms, peak of the first cycle

3 Jun Peak concentration of DA in razor clams:

- central Oregon 86 ppm
- Clatsop beach, northern Oregon 140 ppm
- Long beach, southern Washington 110 ppm

**Second *Pseudo-nitzschia* bloom cycle (10 Jun-3 Jul)**

9 Jun-6 Jul Fourth upwelling event: strongest and coldest event of the year

10 Jun PN bloom decayed; PN = 31% of total diatoms --NO_3_=26µM, SiO_4_=43µM

16 Jun PN bloom rebounding; PN =7% of total diatoms-- NO_3_=21µM, SiO_4_=37µM

16 Jun DA in razor clams declines by ~ 50%; remains stable through August

28 Jun PN cells = 43% of total diatoms --NO_3_=3µM, SiO_4_=5µM

3 Jul PN cells = 56% of total diatoms, peak of the second bloom cycle

Upwelling Weakens in July; dinoflagellate, ciliates and nano-flagellates dominate

15 Jul PN cells = 0% of total diatoms

26 Jul PN cells = 0% of total diatoms

27 Jul-3 Aug Upwelling event resumed

**Third *Pseudonitzschia* bloom cycle (10-26 Aug)**

10 Aug PN cells = 16% of total diatoms

14-23 Aug Upwelling event; NO_3_=<0.6 µM, SiO_4_=<2,2 µM

26 Aug PN cells = 39% of total diatoms
