# Supplementary material for: Initiation and Development of a Toxic and Persistent Pseudo-nitzschia Bloom off the Oregon Coast in Spring/Summer 2015
Source: PLoS One. 2016 Oct 12;11(10):e0163977. doi: 10.1371/journal.pone.0163977 (PMC5061394; doi:10.1371/journal.pone.0163977)
Supplement: S1 Text — (DOCX) [file pone.0163977.s005.docx]

**S1 Text. Descriptions of phytoplankton community changes following S1 Table**

In February and early March, winter diatom blooms developed, with moderately high species richness (Table S1, as below). No PN cells were seen during this bloom. A peak in chlorophyll was noted on March 4; the dominants were *Asterionellopsis glacialis*, *Thalassiosir*a spp., *Chaetoceros* spp., and *Skeletonema costatum*. By the end of March, those previously bloomed species were still abundant, but very few larger PN cells were found and only at the most inner-shelf station NH1. Two weeks after the onset of upwelling season (on April 12), a large diatom bloom formed (April 27) and was featured by multiple species, including PN species, *Skeletonema costatum*, *Asterionellopsis glacialis* and 11 species from the genus of *Chaetoceros*. During the mono-specific PN bloom in May, the overall diatom species richness was reduced greatly. The most abundant diatoms were PN, *Skeletonema costatum*, *Nitzschia*, *Thalassiosira subtilis*, *Detonula pumila* and *Eucampia zodiacus.* With the decrease of PN dominance in mid-June, other diatoms which commonly bloom off Oregon became more abundant and co-dominant. When the highest peak of PN abundance was seen at the end of June (28^th^) and early July (3^rd^), PN dominance increased to ~ 50%; diatom species that bloomed earlier in the year were also prevalent resulting in the second peak of diatom species richness. In July with the collapse of coastal upwelling for nearly one month, dinoflagellates and ciliates (also nanoflagellates) were more abundant than diatoms. Diatom dominance returned in August with a different set of dominants including *Ditylum brightwellii*, *Probosia alata* in mid-August and *Rhizosolenia styliformis* co-dominated the phytoplankton community with PN dominance at the end of August.
